# FGF2 modulates cardiac remodeling in an isoform- and sex-specific manner

**DOI:** 10.1002/phy2.88

**Published:** 2013-09-17

**Authors:** Eyad Nusayr, Doraid Tarek Sadideen, Tom Doetschman

**Affiliations:** 1 Department of Cellular and Molecular Medicine, College of Medicine, College of Science, The University of ArizonaTucson, Arizona; 2 College of Science, The University of ArizonaTucson, Arizona; 3 BIO5 Institute, The University of ArizonaTucson, Arizona

**Keywords:** Cardiac fibrosis, cardiac hypertrophy, cardiac remodeling, FGF2 isoforms, sex

## Abstract

Pathological cardiac hypertrophy and cardiac fibrosis are remodeling events that result in mechanical stiffness and pathophysiological changes in the myocardium. Both humans and animal models display a sexual dimorphism where females are more protected from pathological remodeling. Fibroblast growth factor 2 (FGF2) mediates cardiac hypertrophy, cardiac fibrosis, and protection against cardiac injury, and is made in high molecular weight and low molecular weight isoforms (Hi FGF2 and Lo FGF2, respectively). Although some light has been shed on isoform-specific functions in cardiac pathophysiology, their roles in pathologic cardiac remodeling have yet to be determined. We tested the hypothesis that Lo FGF2 and Hi FGF2 modulate pathological cardiac remodeling in an isoform-specific manner. Young adult male and female mice between 8 and 12 weeks of age of mixed background that were deficient in either Hi FGF2 or Lo FGF2 (Hi KO or Lo KO, respectively) were subjected to daily injections of isoproterenol (Iso) for 4 days after which their hearts were compared to wild-type cohorts. Post-Iso treatment, female Lo KO hearts do not exhibit significant differences in their hypertrophic and fibrotic response, whereas female Hi KO hearts present with a blunted hypertrophic response. In male animals, Lo KO hearts present with an exacerbated fibrotic response and increased *α*-smooth muscle actin protein expression, whereas Hi KO hearts present with a blunted fibrotic response and increased atrial natriuretic factor protein expression Thus, in female hearts Hi FGF2 mediates cardiac hypertrophy, whereas in male hearts Lo FGF2 and Hi FGF2 display an antithetical role in cardiac fibrosis where Lo FGF2 is protective while Hi FGF2 is damaging. In conclusion, cardiac remodeling following catecholamine overactivation is modulated by FGF2 in isoform- and sex-specific manners.

## Introduction

Pathologic cardiac remodeling is commonly associated with decompensated hypertrophy and fibrosis of the myocardium. Both of these events are considered detrimental for normal cardiac function and are common findings in heart disease which is the leading cause of death in the United States (Levy et al. 1990). Understanding the mechanisms that underlie these disease processes will aid in the development of clinical therapeutics to lessen the morbidity and mortality of cardiovascular disease.

Fibroblast growth factor 2 (FGF2), a prototypic member of the FGF family, is encoded by a single gene. However, alternative translation–initiation codons produce various isoforms (Touriol et al. 2003). Low molecular weight FGF2 (Lo FGF2) is an 18 kDa protein translated from a conventional AUG start codon and its 155 amino acid sequence is common to all FGF2 isoforms (Ibrahimi et al. 2004). The high molecular weight (Hi FGF2) isoforms (20.5 and 21 kDa) are produced by starting translation at CUG sites upstream and inframe of the AUG codon (Prats et al. 1989).

In mouse models of cardiac hypertrophy that are induced by pressure overload or adrenergic overstimulation, there is a marked upregulation of *Fgf2* mRNA (Padua and Kardami 1993; Spruill et al. 2008). An upregulation of *FGF2* mRNA was also measured in human patients presenting with ventricular hypertrophy (He et al. 2005). We have previously shown that FGF2 mediates cardiac hypertrophic and fibrotic responses to pressure overload induced by transverse aortic coarctation (Schultz et al. 1999) and to *β* adrenergic stimulation by isoproterenol (Iso) (House et al. 2010). However, these studies either ablated total FGF2 or overexpressed it. The isoform-specific role of endogenous FGF2 in relation to hypertrophy and fibrosis was not examined.

To examine the isoform-specific functions of FGF2, we have generated Lo FGF2 knockout (Lo KO) (Garmy-Susini et al. 2004) and Hi FGF2 knockout (Hi KO) (Azhar et al. 2009) mice using the tag-and-exchange procedure (Askew et al. 1993) which left no residual loxP sites that might interfere with the non-FGF2–related reading frames embedded within the first intron of the gene or with any possible regulatory information. Using Lo KO and Hi KO mice we recently demonstrated that FGF2 isoforms have distinct functional roles in the unstressed heart (Nusayr et al. 2013). At baseline measurements we demonstrated that female Lo KO hearts present with a restrictive filling pattern and thinner walls, whereas male Lo KO hearts display an impaired relaxation filling pattern. Female Hi KO hearts do not differ from their wild type (WT), whereas male Hi KO hearts present with an enhanced systolic function. Thus, the hearts of these mice exhibit systolic, diastolic, and structural deviations from their WT cohorts that are also isoform and sex specific.

Sexual dimorphism in the field of cardiac pathology is observed in both humans and in genetically manipulated mice (Du 2004). Premenopausal women display a lower prevalence of pathological remodeling than their male counterparts even after correcting for risk factors (Babiker et al. 2002). In this study both male and female animals were treated with Iso, known to cause cardiac hypertrophy and fibrosis in mice (Nowak et al. 1975), in order to discern sex-specific pathologies associated with the deletion of specific FGF2 isoforms. The use of Iso is clinically relevant as overactivation of adrenergic signaling is an independent risk factor for cardiac morbidity and mortality (Osadchii 2007). In addition, sympathetic overactivation is associated with other cardiac remodeling stimuli such as pressure overload (Siri 1988) and volume overload (Willenbrock et al. 1997).

This is the first report on the role of endogenous FGF2 isoforms in cardiac remolding, and our results demonstrate that following Iso treatment male Lo KO hearts display an exacerbated fibrotic response and induction of *α*-smooth muscle actin (*α*-SMA), whereas male Hi KO hearts exhibit attenuated fibrosis and induction of atrial natriuretic factor (ANF). In the female group, Hi KO hearts present with a blunted hypertrophic response to Iso. Thus, the results provide novel evidence that FGF2 isoforms have nonredundant mechanistic functions in cardiac remodeling that are modulated by sex.

## Material and Methods

### Animals

WT, Lo KO (*Fgf2*^*tm2Doe*^/J), and Hi KO (*Fgf2*^*m3Doe*^/J) FGF2 mice have been described previously (Garmy-Susini et al. 2004; Azhar et al. 2009) and were bred on a mixed Black Swiss (50%)/129 (50%) background. The mice were 8–12 weeks of age and were housed in a specific pathogen free vivarium at the University of Arizona which is *Helicobacter* and Norovirus free. Animals in the facility are housed in microisolator cages in ventilated cage racks with automatic watering. This study was approved by IACUC #09-037 entitled “Cardiac Hypertrophy Studies”. Genotyping of these mice was performed by the University of Arizona Genetics Core (UAGC) in the University of Arizona's Arizona Research Laboratory (ARL). Five to 12 mice from each sex/genotype group were used for each data point (Tables 1 and 2).

**Table 1:** Female gravimetric and morphometric measurements

	Pre-Iso			Post-Iso			Pre- vs. Post-Iso *P*-value		
			
	WT	Lo KO	Hi KO	WT	Lo KO	Hi KO	WT	Lo KO	Hi KO
*n*	9	9	9	12	12	12			
Heart/Body	4.9 ± 0.2	4.2 ± 0.1	4.9 ± 0.1	5.1 ± 0.2	4.6 ± 0.2#	5.3 ± 0.2	NS	0.010	NS
*n*	7	5	5	6	6	6			
Cardiomyocyte area (μm^2^)	178 ± 5	194 ± 8	181 ± 3	235 ± 8#	246 ± 9#	194 ± 3#	0.001	0.002	0.015

Values are means ± SEM. *n*, number of mice; H/B, heart (mg)/body (g); NS, not significant.

# Statistical significance versus Pre-Iso cohorts.

**Table 2:** Male gravimetric and morphometric measurements

	Pre-Iso			Post-Iso			Pre- vs. Post-Iso *P*-value		
			
	WT	Lo KO	Hi KO	WT	Lo KO	Hi KO	WT	Lo KO	Hi KO
*n*	6	6	6	11	11	11			
H/B (ratio)	4.7 ± 0.3	4.5 ± 0.3	4.8 ± 0.2	5.2 ± 0.2	5.1 ± 0.2	5.4 ± 0.1*	NS	NS	0.047
*n*	5	5	5	6	6	6			
Cardiomyocyte area (μm^2^)	191 ± 12	210 ± 9	205 ± 4	247 ± 12*	250 ± 8*	243 ± 13*	0.010	0.009	0.035

Values are means ± SEM. *n*, number of mice; H/B, heart (mg)/body (g); NS, not significant.

* Statistical significance versus Pre-Iso cohorts.

To induce hypertrophy and fibrosis, subcutaneous injections of Iso (2 μg/g/day) were used (Hohimer et al. 2005) for 4 consecutive days and the hearts collected on the 5th day. The animals were divided into two groups. One group was given Iso and designated as the post-Iso group; the other group was not given Iso and was designated as the pre-Iso group.

The hearts from both groups were fixed and preserved in 1:10 buffered formalin before being embedded in paraffin blocks. The paraffin embedded hearts were sectioned at the midventricular plane at a thickness of 2 microns. Fixed heart sections underwent a protocol of deparaffinization that involved two changes of xylene, 100% ethanol, 95% ethanol, 70% ethanol, and water.

### Wheat germ agglutinin staining

Heart sections were stained with wheat germ agglutinin (WGA) conjugated to the rhodamine derivative TRITC (Sigma St Louis, MO). The sections were boiled in a microwave oven for 3 min before incubation in the dark with TRITC-WGA for 40 min at 37°C.

The pre-Iso female subgroup consisted of sections representing seven WT, five Lo KO, and five Hi KO animals, whereas the male subgroup consisted of sections representing five WT, five Lo KO, and five Hi KO animals. The post-Iso female subgroup consisted of sections representing six WT, six Lo KO, and six Hi KO animals, whereas the male subgroup consisted of sections representing six WT, six Lo KO, and six Hi KO animals.

### Trichrome staining

A trichrome stain kit was used (modified Masson's) (ScyTek laboratories, Logan, UT) and applied to heart sections using a standard protocol. Briefly, two equal parts of Weigert's solution were mixed and applied to sections, followed by Biebrich scarlet/acid Fuchsin solution. To differentiate, phosphomolybdic/phosphotungstic acid solution was used. Aniline blue was then applied and the excess was washed away with 1% acetic acid.

The pre-Iso female subgroup consisted of sections representing four WT, four Lo KO, and four Hi KO animals, whereas the male subgroup consisted of sections representing five WT, six Lo KO, and five Hi KO animals. The post-Iso female subgroup consisted of sections representing seven WT, six Lo KO, and 11 Hi KO animals, whereas the male subgroup consisted of sections representing eight WT, eight Lo KO, and eight Hi KO animals.

### Immunohistochemistry

Immunohistochemistry (IHC) (Dako, Carpentria, CA) on heart sections was done using the manufacturer's protocol. The primary antibodies used were antisera for collagen I at 1:500 (Col I) (Abcam, Cambridge, MA), *α*-SMA at 1:250 (Abcam), and ANF at 1:200 (Abcam).

The Col I pre-Iso female subgroup consisted of sections representing four WT, four Lo KO, and five Hi KO animals, whereas the male subgroup consisted of sections representing five WT, six Lo KO, and five Hi KO animals. The post-Iso female subgroup consisted of sections representing six WT, six Lo KO, and six Hi KO animals, whereas the male subgroup consisted of sections representing six WT, six Lo KO, and six Hi KO animals.

The *α*-SMA pre-Iso female subgroup consisted of sections representing four WT, four Lo KO, and five Hi KO animals, whereas the male subgroup consisted of sections representing five WT, six Lo KO, and five Hi KO animals. The post-Iso female subgroup consisted of sections representing six WT, five Lo KO, and six Hi KO animals, whereas the male subgroup consisted of sections representing six WT, six Lo KO, and six Hi KO animals.

The ANF pre-Iso female subgroup consisted of sections representing four WT, four Lo KO, and four Hi KO animals, whereas the male subgroup consisted of sections representing six WT, five Lo KO, and five Hi KO animals. The post-Iso female subgroup consisted of sections representing six WT, six Lo KO, and six Hi KO animals, whereas the male subgroup consisted of sections representing six WT, six Lo KO, and six Hi KO animals.

### Imaging

All images were captured using a Zeiss-Image M1 microscope. To measure cardiomyocyte area, fibrotic area, tissue area, and expression of remodeling markers, the Zeiss-Axiovision software (version 4.7.2, Carl Zeiss, Jena, Germany) was used according to developer's protocols.

The extent of cardiomyocyte enlargement was examined in the subendocardial layer (Pandya et al. 2006) of the left ventricle (LV)-free wall using TRITC-WGA to stain the cell wall of cardiomyocytes and measure their dimensions. Six nonoverlapping microscopic images of the free LV wall from each heart were made. At least 200 cardiomyocytes were measured from each heart.

### Statistical analysis

Two-sided student's *t*-test with unequal variance was used to evaluate the data. A difference was considered significant when *P* < 0.05. In the panels of barographs, the height of the bar represents the mean while the error bar represents the standard error of the mean.

## Results

### Heart weight differences in Hi and Lo KO animals are sex and isoform specific

**Figure 1:**
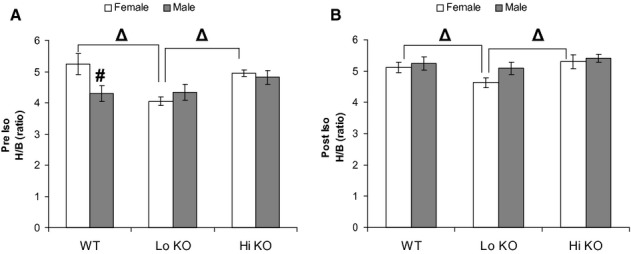
Pre-Isoproterenol (Iso) and Post-Iso assessments of the gravimetry of female (white bar) and male (gray bar) wild-type (WT), low molecular weight knockout (Lo KO), and high molecular weight knockout (Hi KO) hearts. Note the smaller normalized hearts mass of female Lo KO hearts which is consistent with hypoplasia. (A) H/B, heart (mg)/body (g) ratio in pre-Iso group. (B) H/B in post-Iso group. (Δ); Statistical significance across female genotypes. Each bar represents data from 6 to 12 animals; Graphs are presented as means ± SEM for each genotype/sex and significance noted when *P* < 0.05.

To grossly assess the interaction of isoform deletions and sex on the hearts, we measured heart weights in pre- and post-Iso groups and normalized them to body weight (Tables 1, 2; and Fig. 1). As cardiac hypertrophy involves an increase in cell volume and thus cell mass (Lorell and Carabello 2000), heart-to-body weight ratio (H/B) is frequently used as an indicator for the presence or absence of cardiac hypertrophy.

Iso had the effect of increasing H/B for all the groups; however, this increase was only statistically significant in the Lo KO females (Table 1) and the Hi KO males (Table 2). This indicates that the effect of the Iso regimen was too acute to achieve a robust increase in H/B in all groups.

Prior to Iso treatment, the hearts from Lo KO females have a significantly smaller H/B when compared to either Hi KO female hearts or WT female hearts (Fig. 1A). Treatment with Iso does not eliminate this difference, and the female Lo KO H/B remains significantly smaller (Fig. 1B). Similar differences in H/B do not occur in males whether they are from the pre- or post-Iso groups (Fig. 1A and B). This indicates that Lo FGF2 is necessary for normal cardiac growth in the female mouse.

### Hypertrophic response differences in Hi and Lo KO hearts are sex and isoform specific

**Figure 2:**
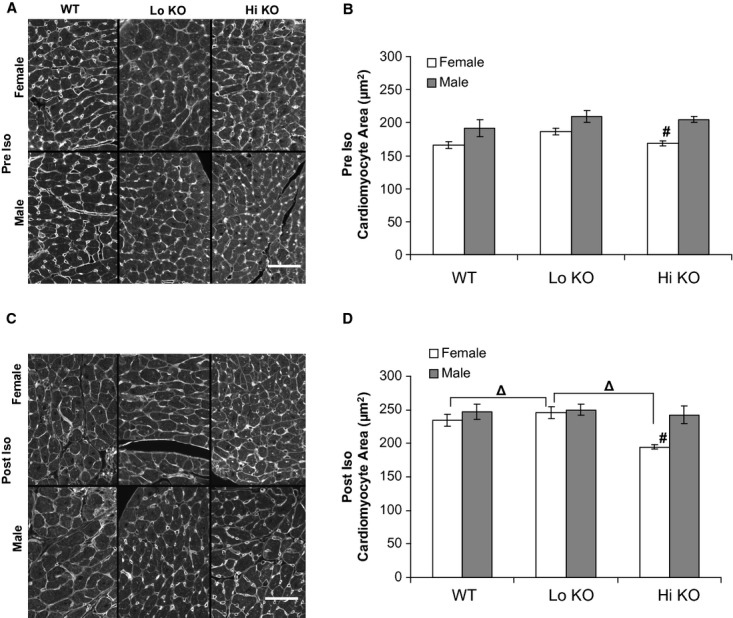
Pre-Iso and Post-Iso assessments of the morphometry of female and male WT, Lo KO, and Hi KO hearts. Note the smaller cardiomyocyte area of post-Iso female Hi KO hearts which is consistent with a blunted hypertrophic response. (A) TRITC-WGA staining of representative sections from pre-Iso WT, Lo KO, and Hi KO hearts. (B) Quantification of cardiomyocyte area from pre-Iso sections. (C) TRITC-WGA staining of representative sections from pre-Iso WT, Lo KO, and Hi KO hearts. (D) Quantification of cardiomyocyte area from post-Iso sections. (Δ); Statistical significance across female genotypes. (#); Statistical significance across sex for same genotype. Each bar represents data from five to seven animals and at least six nonoverlapping images from the subendocardial layer of the left ventricle (LV); Graphs are presented as means ± SEM; (white bar) female and (gray bar) male. Significance noted when *P* < 0.05. Scale bar = 50 μm.

Cardiac hypertrophy is the increase in heart mass due to an increase in cardiomyocyte volume (Lorell and Carabello 2000). Using sections stained with TRITC-WGA (Fig. 2A and C), we measured cardiomyocyte area in pre- and post-Iso groups to assess the hypertrophic response. WGA is a lectin that preferentially stains cell membranes in the myocardium and is routinely used to evaluate hypertrophy by measuring cardiomyocyte area to infer changes in cardiomyocyte size.

The pre-Iso group did not display any significant differences in cardiomyocyte area (Fig. 2A and B) with the exception of female Hi KO cardiomyocytes which are significantly smaller than their male counterparts (Fig. 2B).

Post-Iso females present with a significant increase in cardiomyocyte area; however, this increase is attenuated in Hi KO hearts as the area for WT, Lo KO, and Hi KO cardiomyocytes increased by 31%, 30%, and 7%, respectively (Table 1).

In post-Iso males, the area for WT, Lo KO, and Hi KO hearts increased by 29%, 27%, and 19%, respectively (Table 2). In the female post-Iso group, the Hi KO cardiomyocytes are significantly smaller in area than both their WT and Lo KO cohorts (Fig. 2C and D). These data demonstrate that *β*-adrenergic activation causes cardiomyocyte hypertrophy in both sexes regardless of genotype. However, the response in female Hi KO hearts is blunted even though their cardiomyocyte area prior to treatment is comparable to WT hearts, indicating an important role for Hi FGF2 in mediating cardiac hypertrophy in female hearts. The absence of this phenotype in Hi KO male hearts suggests that Hi FGF2 is required for cardiomyocyte hypertrophy in female but not male hearts.

### Fibrotic response differences in Hi and Lo KO hearts are sex and isoform specific

**Figure 3:**
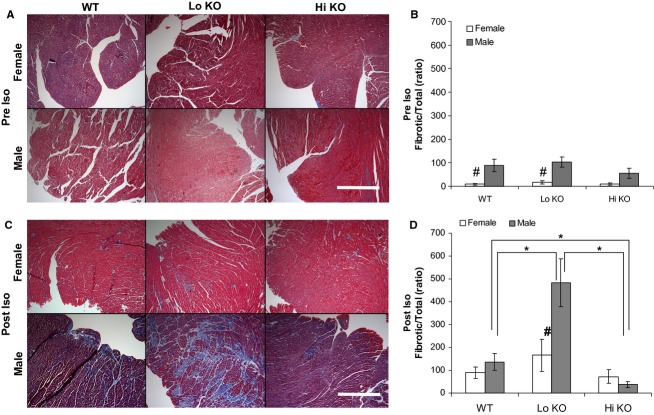
Pre-Iso and Post-Iso assessments of the fibrotic area in female and male WT, Lo KO, and Hi KO hearts. Note the larger fibrotic area of post-Iso male Lo KO hearts and the smaller fibrotic area of post-Iso male Hi KO hearts. (A) Pre-Iso representative image of sections stained with Trichrome from male and female WT, Lo KO, and Hi KO hearts. (B) Pre-Iso quantification of fibrotic area normalized to total tissue area. (C) Post-Iso representative image of sections stained with trichrome from male and female WT, Lo KO, and Hi KO hearts. (D) Post-Iso quantification of fibrotic area normalized to total tissue area. (*) Statistical significance across male genotypes. (#) Statistical significance across sex for same genotype. Each bar represents data from five to eight animals and at least six nonoverlapping images from each LV. Scale bar = 500 μm. Graphs are presented as means*10^4^ ± SEM; (white bar) female and (gray bar) male. Significance noted when *P* < 0.05.

In order to assess the interaction of sex and isoform deletions on the fibrotic process we stained heart sections with trichrome, which stains collagen and fibrotic areas blue while nonfibrotic tissue stains red (Fig. 3A and C). We measured the fibrotic areas and normalized the measurements to total tissue area; both red and blue (Fig. 3B and D).

Pre-Iso male and female heart sections from WT, Lo KO, and Hi KO animals show no significant differences in fibrous area measurements within the same-sex group (Fig. 3A and B). Of interest, the pre-Iso WT and Lo KO male hearts have significantly larger fibrous areas when compared to their female counterparts but not the male Hi KO hearts (Fig. 3B).

Post-Iso, the male Lo KO hearts show an exaggerated fibrotic response to Iso where the fibrotic area is threefold more than in WT hearts. On the other hand, male Hi KO hearts exhibit a blunted fibrotic response following Iso treatment which was half of that measured in WT controls (Fig. 3D). Similar fibrotic differences do not appear in female hearts post-Iso treatment. These data demonstrate that Lo FGF2 protects male hearts from excessive fibrosis following *β*-adrenergic stimulation. This function of Lo FGF2 is not evident in female hearts and is thus sex specific.

### Induction of remodeling in Hi and Lo KO hearts is sex and isoform specific

**Figure 4:**
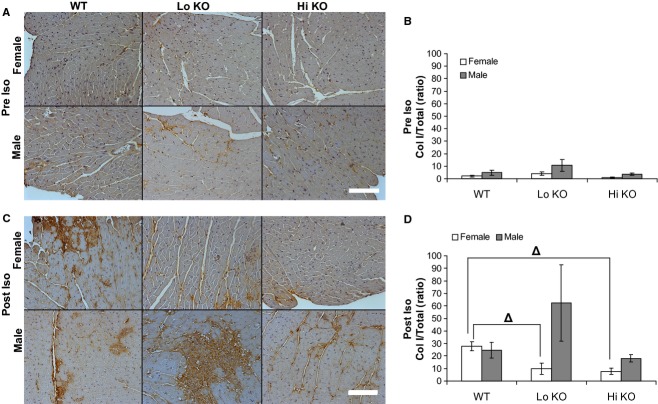
Pre-Iso and Post-Iso assessment of the collagen I (Col I) expression in female and male WT, Lo KO, and Hi KO hearts. Note that both post-Iso Lo KO and Hi KO female hearts display a blunted expression of Col I. (A) Pre-Iso representative image of sections stained for Col I from male and female WT, Lo KO, and Hi KO hearts. (B) Pre-Iso quantification of Col I normalized to total tissue area. (C) Post-Iso representative image of sections stained for Col I from male and female WT, Lo KO, and Hi KO hearts. (D) Post-Iso quantification of Col I area normalized to total tissue area. (Δ) Statistical significance across female genotypes. Each bar represents data from four to six animals and at least six nonoverlapping images from each LV. Scale bar = 100 μm. Graphs are presented as means*10^4^ ± SEM; (white bar) female and (gray bar) male. Significance noted when *P* < 0.05.

**Figure 5:**
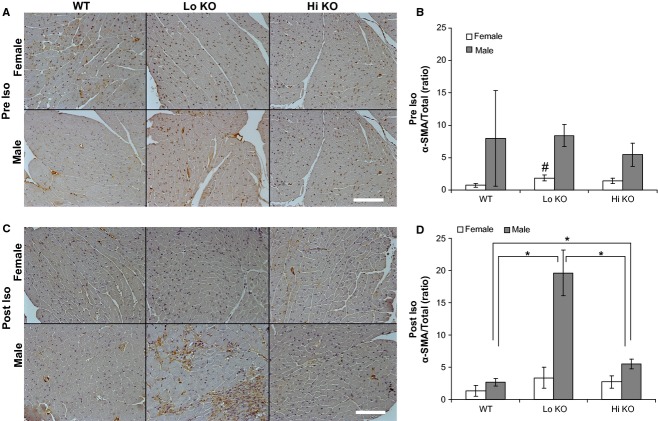
Pre-Iso and Post-Iso assessments of the *α*-smooth muscle actin (*α*-SMA) expression in female and male WT, Lo KO, and Hi KO hearts. Note that post-Iso Lo KO male hearts display an exaggerated expression of *α*-SMA. (A) Pre-Iso representative image of sections stained for *α*-SMA from male and female WT, Lo KO, and Hi KO hearts. (B) Pre-Iso quantification of *α*-SMA normalized to total tissue area. (C) Post-Iso representative image of sections stained for *α*-SMA from male and female WT, Lo KO, and Hi KO hearts. (D) Post-Iso quantification of *α*-SMA area normalized to total tissue area. (*) Statistical significance across male genotypes. (#) Statistical significance across sex for same genotype. Each bar represents data from four to six animals and at least six nonoverlapping images from each LV. Scale bar = 100 μm. Graphs are presented as means*10^4^ ± SEM; (white bar) female and (gray bar) male. Significance noted when *P* < 0.05.

**Figure 6:**
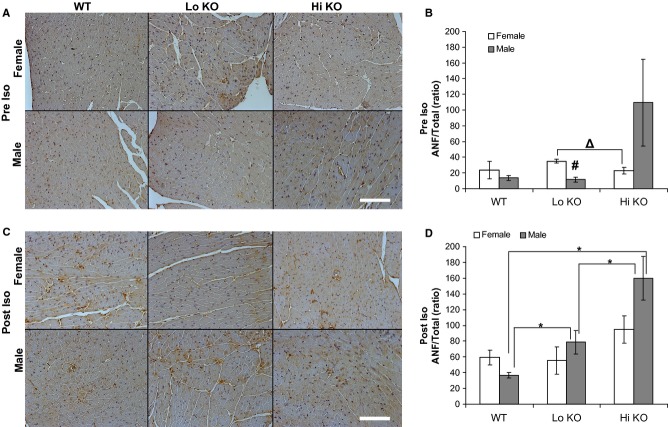
Pre-Iso and Post-Iso assessments of the atrial natriuretic factor (ANF) expression in female and male WT, Lo KO, and Hi KO hearts. Note that post-Iso Hi KO male hearts display an exaggerated expression of ANF. (A) Pre-Iso representative image of sections stained for ANF from male and female WT, Lo KO, and Hi KO hearts. (B) Pre-Iso quantification of ANF normalized to total tissue area. (C) Post-Iso representative image of sections stained for ANF from male and female WT, Lo KO, and Hi KO hearts. (D) Post-Iso quantification of ANF area normalized to total tissue area. (*) Statistical significance across male genotypes. (Δ) Statistical significance across female genotypes. (#) Statistical significance across sex for same genotype. Each bar represents data from four to six animals and at least six nonoverlapping images from each LV. Scale bar = 100 μm. Graphs are presented as means*10^4^ ± SEM; (white bar) female and (gray bar) male. Significance noted when *P* < 0.05.

Using IHC we assayed the expression of known remodeling markers in order to assess the interaction of sex- and isoform-specific deletions on their expression. Using antibodies we measured the expression of Col I (Fig. 4), *α*-SMA (Fig. 5), and ANF (Fig. 6) on cardiac tissue sections, and the measurements were normalized to total tissue area.

The expression of Col I trends to be higher in male Lo KO hearts pre-Iso (Fig. 4A) and post-Iso (Fig. 4C) treatment; however, this trend is not statistically significant (Fig. 4B and D). Post-Iso, the female Lo KO and Hi KO hearts display a blunted expression of Col I when compared to their WT counterparts which have threefold more expression of Col I. (Fig. 4D). Col I is known to be expressed after a few days of cardiac stress (Masson et al. 1998). These results indicate that 4 days of Iso treatment are not sufficient to induce significant Col I production in our experimental groups and that both isoforms have a role in female cardiac extracellular matrix (ECM) remodeling following Iso treatment. The post male-group expression profile of Col I has a trend that is comparable to the levels of trichrome staining. However, the trend is not statistically significant.

The expression of *α*-SMA in pre-Iso hearts (Fig. 5A) is not significantly different across the group except for its expression in male Lo KO hearts which is significantly higher than in their female counterparts (Fig. 5B). Post-Iso (Fig. 5C), we note that male Lo KO hearts exhibit a sevenfold increase over WTs in *α*-SMA expression. The male Hi KO hearts display a twofold increase in *α*-SMA over WT. Similar phenotypic differences do not appear in female hearts post-Iso treatment (Fig. 5D). The level of induction of *α*-SMA in male Lo KO hearts as compared to female Lo KO hearts indicates that Lo FGF2 suppresses *α*-SMA in a sex-specific manner. *α*-SMA is a marker for myofibroblasts which are specialized fibroblasts that contain a large amount of actin filaments, “basal lamina”, and *α*-SMA, which makes them resemble smooth muscle cells. They also have been implicated as the primary producer of collagen during fibrosis and the driving force behind scar contraction (Takeda and Manabe 2011). The higher expression of *α*-SMA in the post-Iso male Lo KO hearts indicates a larger myofibroblast population, which could be responsible for the exaggerated fibrosis these hearts display. Lo FGF2 could therefore counteract myofibroblast induction.

The expression of ANF in the pre-Iso group (Fig. 6A) is higher in female Lo KO hearts than in both their male counterparts and in female Hi KO hearts (Fig. 6B). Pre-Iso male Hi KO hearts trend to higher ANF expression. Post-Iso (Fig. 6C), we note that Hi KO males have fourfold more ANF expression than do their WT counterparts, and twofold more ANF expression than their Lo KO counterparts. Similar phenotypic differences do not occur in female hearts after Iso treatment (Fig. 6D). As ANF is induced in male Hi KO hearts, this suggests that Hi FGF2 suppresses ANF production in a sex-specific fashion.

## Discussion

This study provides novel evidence on the interaction of sex and endogenous FGF2 isoforms as modulators of cardiac remodeling through hypertrophy and fibrosis. Lo FGF2 signaling is necessary in the male heart only to ameliorate the fibrotic response induced by *β*-adrenergic stress, whereas Hi FGF2 is necessary only in female hearts for mediating the hypertrophic response. Hence, we demonstrate that Lo FGF2 and Hi FGF2 have nonredundant roles in cardiac remodeling which are sex specific (Table 3).

In a recent baseline physiological study, we demonstrated that FGF2 has isoform- and sex-specific roles in mouse hearts. Changes in heart geometry, systolic, and diastolic functions were manifestations of FGF2-isoform–specific ablation in a sex-specific manner. In this study we stressed the hearts with *β*-adrenergic stimulation using Iso. Under this condition Lo KO and Hi KO hearts present with differential phenotypes in their fibrotic and hypertrophic responses that are also sex specific. Our results provide evidence that Lo FGF2 protects the heart from a fibrotic response in male animals only. In female animals, the production of Hi FGF2 is required to convey the hypertrophic response following Iso treatment. The results are consistent with an antagonistic effect of Hi FGF2 on Lo FGF2 protection which is associated with an increased expression of ANF.

The form of cardiac stress we used here is the *β*-adrenergic agonist Iso which causes cardiomyocyte hypertrophy and fibrosis in mice by acting on the *β* 1 and *β* 2 receptors (Nowak et al. 1975). On the physiologic level, Iso increases myocardial contractility and heart rate by stimulating Beta-adrenergic receptors (*β*-ARs). At the same time, peripheral vascular resistance is lowered by the action of Iso on *β*-ARs (Dixon et al. 1979). Stimulation with adrenergic agonists can also upregulate FGF2 protein and message expression (Padua and Kardami 1993).

The isoforms of FGF2 are expressed in fixed molar ratios that are dependent on tissue type. These ratios are translationally regulated as overexpression of the human FGF2 gene in mice does not alter these ratios in the human FGF2 protein products (Coffin et al. 1995). Hi FGF2 and Lo FGF2 can potentially associate reciprocally, modulating each others' biological activity depending on their relative ratios and/or localization (Pintucci et al. 2005; Quarto et al. 2005). FGF2 isoforms are localized differentially and display different gene expression profiles (Quarto et al. 2005). In response to ischemia/reperfusion (I/R) injury only Lo FGF2 can be released from cardiac cells further indicating the intracrine localization of Hi FGF2 (Liao et al. 2010). Exported Lo FGF2 binds to the FGF receptor (FGFR) extracellularly and its binding is modulated by nonsignaling heparin/heparan sulfate proteoglycans that are subsequently involved in the intracellular processing of FGF2 (Sperinde and Nugent 2000).

Our collaborators' studies using transgenic and Lo and Hi KO strains provided in vivo evidence that Hi FGF2 antagonizes Lo FGF2 function; and whereas Lo FGF2 causes phosphorylation/activation of FGFR1, Hi FGF2 inhibits it. Phenotypically, this was supported by the deleterious effect of overexpressing Hi FGF2 versus the protective effect of overexpressing Lo FGF2 on the extent of I/R injury (Liao et al. 2007, 2010). This differential role of FGF2 isoforms is also seen in this study. We demonstrate that deletion of Lo FGF2 enhances the fibrotic response in male hearts, whereas deleting Hi FGF2 attenuates it. It was previously demonstrated that the cardioprotective effect of Lo FGF2 includes an antiapoptotic function (Liao et al. 2007, 2010), whereas Hi FGF2 is a proapoptotic protein that induces chromatin condensation and cell death through mitochondrial involvement and extracellular signal-regulated kinase activation in human embryonic kidney cells (Ma et al. 2007). This is consistent with our finding that the male Lo KO heart presents with a more severe phenotype where increased cell death would result in a greater degree of reparative fibrosis (Weber et al. 1989). We also know from our collaborators' study that deletion of Hi FGF2 improves the outcome following I/R (Liao et al. 2010). In the results presented here, the deletion of Hi FGF2 in male hearts results in the attenuation of the fibrotic response to Iso treatment which could indicate that in the absence of the antagonistic effect of Hi FGF2, Lo FGF2 signaling through FGFR is enhanced which in turn would decrease apoptosis and subsequently reduce reparative fibrosis.

Sexual dimorphism in the context of cardiac pathology is a recognized phenomenon in both humans and animal models. The incidence of LV hypertrophy and coronary artery disease is less in premenopausal women (Babiker et al. 2002). For the most part, estrogen is associated with less morbidity, whereas male hormone contributions are considered detrimental (Du 2004). Estrogen can also modulate FGF2 isoforms as activation of the estrogen pathway in endothelial cells results in an isoform switch from mostly Lo to predominantly Hi FGF2 (Garmy-Susini et al. 2004). To date, there is no report on the interaction of FGF2 and sex in cardiac pathology. The fibrotic response to Iso in Lo and Hi KO hearts demonstrates sex-specific roles of FGF2 isoforms. We find that only male hearts display an isoform-dependent differential fibrotic response following Iso treatment. Consistently, only post-Iso male hearts display significant differences in *α*-SMA and ANF expression. Hence, we note an interaction between sex and FGF2 isoform deletion that results in distinct responses to *β*-adrenergic stimulation.

Because of the significant effect of sex on cardiac phenotype, we examined both male and female animals separately to discern sex-specific functions of FGF2 isoforms under cardiac stress. The results presented here are consistent with previous observations of male susceptibility to cardiac pathology: Iso treatment of Lo KO males resulted in a fibrotic phenotype which indicates massive cell loss in the myocardium, whereas the female Lo KO hearts do not significantly differ from their WT cohorts in their hypertrophic and fibrotic response. Hence, males are more prone to cardiac damage due to Lo FGF2 deletion. Previous studies demonstrated that estrogen induces FGFR1 expression and this induction is mediated by Hi FGF2 (Garmy-Susini et al. 2004). It is possible that a protective effect is mediated by other growth factors signaling through cross talk with FGFR1 (Miraoui and Marie 2010) saving the Lo KO female hearts from the fibrotic phenotype observed in the male Lo KO hearts. In addition, in the Hi KO female hearts the induction of FGFR1 by estrogen is blunted and this reduced FGFR1 expression could be responsible for the blunted hypertrophic phenotype observed in these hearts. To date, there is no information on the relationship between FGF2 signaling and androgens, which warrants a castration model for future investigations.

FGF2 is a potent regulator of cardiomyocyte proliferation (deAlmeida and Sedmera 2009) and our examination of heart weight and cardiomyocyte area reveals that pre-Iso and post-Iso female Lo KO hearts are smaller in weight, but similar in cardiomyocyte area when compared to their WT cohorts. This indicates that the female Lo KO is hypoplastic and that Lo FGF2 is required for normal cardiomyocyte proliferation in female hearts.

In vitro studies indicate that ANF can inhibit the hypertrophy of cardiomyocytes and the proliferation of cardiac fibroblasts (Calderone et al. 1998). In addition, in a mouse model of dilated cardiomyopathy, it was shown that endogenous ANF has a protective effect on the heart (Yasuno et al. 2009). Our results show that the expression on ANF is relatively high in male Hi KO hearts post-Iso treatment and there is a trend of higher ANF expression in pre-Iso Hi KO hearts which may presage why the male Hi KO hearts have a protective phenotype that is resistant to fibrosis; namely, the absence of Hi FGF2 would allow for more ANF production and, as a result, more protection against fibrosis. This would be consistent with the antagonistic role of Hi FGF2 such that in its absence the remaining Lo FGF2 would be responsible for increased ANF production and resistance to fibrosis in the male Hi KO hearts.

In humans, nonmyocyte cells (NMCs) comprise 25% of the myocardial volume but 60–70% of the cell count. Most NMCs are fibroblasts, whereas endothelial and smooth muscle cells represent a smaller population. In addition, cells specialized for immunological roles are also present in the myocardium (e.g., macrophages, monocytes, neutrophils, and lymphocytes) which can secret interleukins, cytokines, and growth factors that can drastically change the behavior of target cells (Takeda and Manabe 2011). In order to understand the mechanisms of cardiac fibrosis and hypertrophy, it is necessary to consider the roles of both cardiomyocytes and NMCs. As FGF2 is expressed in both cardiomyocytes and NMCs (Liao et al. 2010), it is likely that FGF2 isoforms have differential functions in those two cell populations. Hence, the fibrosis seen in our Lo KO hearts could result from dysregulation of NMC maintenance of ECM, and the blunted hypertrophy seen in Hi KO hearts results from dysregulation of cardiomyocyte protein synthesis. FGF2 isoforms may also differentially modulate the cross talk between cardiomyocytes and NMCs (Pellieux et al. 2001).

An immunoinflammatory response has been implicated in cardiac hypertrophy and fibrosis (Takeda and Manabe 2011). In the fibroblast NIH-3T3 cell line, expression of the nuclear 24 kDa isoform of FGF2 was associated with induction of interleukin-6 (IL6) gene expression (Delrieu et al. 1998), whereas treatment with exogenous 18-kDa isoform of FGF2 resulted in IL-6 downregulation (Delrieu et al. 1998). The cytokine IL6 is a known mediator of the hypertrophic response (Molkentin and Dorn 2001). FGF2 isoforms could be acting through a number of signaling mechanisms to exert their effect. This includes the cytokines of the immunoinflammatory response as modulators of cardiac hypertrophy and fibrosis. Our Lo KO and Hi KO mice provide a useful tool for exploring this possibility. In vitro stimulation of cardiomyocytes with Iso is reported to elicit the cytokine Transforming growth factor-beta1 (TGFβ1) signaling as a mediator of hypertrophy (Schluter et al. 1995) which in turn can trigger myofibroblast transformation (Takeda and Manabe 2011). The fibrotic phenotype and increased expression of the myofibroblast marker *α*-SMA in male Lo KO hearts are consistent with a role for Lo FGF2 in suppressing profibrotic cytokine production, such as TGFβ1, whereas the blunted hypertrophy of female Hi KO hearts suggests that Hi FGF2 might mediate hypertrophy through induction of prohypertrophic cytokines.

The hypertrophic response following Iso treatment does not follow the degree and timing of cardiomyocyte loss, unlike myocardial fibrosis which is closely related to the necrosis of heart cardiomyocytes which in turn follows the dose of administered Iso (Benjamin et al. 1989). This suggests that Iso-induced hypertrophy is a separate event from Iso-induced fibrosis. Here, we show that fibrosis and hypertrophy can be unlinked through FGF2-isoform–specific deletion. This is exemplified in Hi KO male hearts where Iso treatment causes minimal fibrosis and significant levels of hypertrophy.

**Figure 7:**
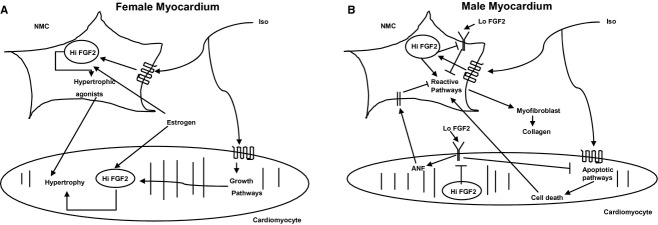
FGF2 isoforms and sex dimorphism. A model of the mechanism by which Lo and Hi FGF2 modulate cardiac remodeling. (A) In the female, myocardium Iso signals through the *β*-AR surface receptors and induces growth pathways in the cardiomyocyte and the production of hypertrophic agonists in the nonmyocyte cells (NMCs) such as fibroblasts, myofibroblasts, endothelial cells, and immune cells. These events are mediated by intracrine Hi FGF2 and estrogen. (B) In the male, myocardium Iso signals through the *β*-AR surface receptors and induce apoptotic pathways in the cardiomyocyte and reactive pathways in the NMCs that lead to cardiac fibrosis. These events are ameliorated by paracrine Lo FGF2 signaling through FGFR, which protects the cardiomyocyte from apoptosis. It also induces ANF, which in turn signals through its receptor to inhibit the reactive pathways in NMCs. Hi FGF2 antagonizes Lo FGF2 at the receptor level in both cardiomyocytes and NMCs. (↑) Stimulatory signal. (⊥) Inhibitory signal.

Our results indicate a sexual dimorphic function of FGF2 isoforms in mediating cardiac protection and cardiac remodeling. The results from female hearts propose a model where Hi FGF2 is downstream of *β*-AR signaling in both cardiomyocytes and NMCs. In cardiomyocytes, the growth pathways (such as MAPK pathway) are activated downstream of Hi FGF2 to induce hypertrophy. Furthermore, Iso acting on NMCs could also be inducing prohypertrophic agonists (such as cytokines) that are secreted to act on cardiomyocytes (Fig. 7A). Estrogen signals in both cardiomyocytes and NMCs upstream of the Hi FGF2-dependent growth pathways. Pellieux and colleagues demonstrated in vitro that WT NMCs are critical to the hypertrophic response following Angiotensin II stimulation, whereas endogenous FGF2 expression in cardiomyocytes is not (Pellieux et al. 2001). Whether this is the case after Iso treatment remains to be tested.

On the other hand, the results from male hearts are consistent with a model where Lo FGF2 counteracts the apoptotic pathways elicited by *β*-AR activation in cardiomyocytes in part through induction of ANF (Fig. 7B). This antiapoptotic action is antagonized by Hi FGF2 at the FGFR level. Iso stimulation of NMCs in addition to cell death induces reactive pathways in these cells (such as cytokine induction) to promote myofibroblast transformation and collagen deposition. In the absence of Hi FGF2, the ability of Lo FGF2 to induce ANF in cardiomyocytes is enhanced. This ANF can also act on NMCs to inhibit the reactive pathways that would otherwise lead to cardiac fibrosis. Also, in NMCs the FGF2 isoforms could be acting on the reactive pathways antithetically, where Lo FGF2 is suppressing these pathways and inhibiting the fibrotic process, and Hi FGF2 is either mediating the Iso-induced reactive pathways or inhibiting Lo FGF2 function.

The results of this study provide a basis to explore the mechanistic aspects of FGF2-isoform functions in cardiac remodeling. In particular, the signaling pathways of endogenous Hi FGF2 and its interaction with Lo FGF2 and its signaling pathway are even less well understood than that of Lo FGF2. The interaction between sex hormones and FGF2 isoforms to produce the sex-specific phenotypes observed in this study remains to be investigated. Furthermore, in the context of the heart alone it is still unexamined how the isoforms modulate physiological hypertrophy or pathological remodeling in response to pressure or volume overload or renin–-angiotensin system activation. Of interest is how these isoforms regulate ECM composition and cross talk between the cellular constituents of the myocardium. With our Lo and Hi KO mice we possess the necessary tools to address these and other questions.

The Iso model of inducing hypertrophy utilized in this study is clinically relevant as sympathetic overactivation is detected in patients with chronic pressure overload and pathological cardiac hypertrophy. Furthermore, in rodent models of pressure overload-induced hypertrophy there is a marked increase in circulating catecholamines. Hence, it is likely that the protective functions of Lo FGF2 versus the adverse role of Hi FGF2 would apply to other models of pathological cardiac remodeling.

The results of this study provide in vivo evidence that sex-specific cardiac remodeling utilizes two distinct molecular mechanisms through FGF2 isoforms. Furthermore, they indicate that fibrosis and hypertrophy can be unlinked through manipulating FGF2 isoform expression. This is pertinent to clinical interests as fibrosis is partly a result of cardiomyocyte loss and in itself leads to a stiffer myocardium, arrhythmia, and reduced diffusion of waste products and nutrients. The fact that male Hi KO mice develop less fibrosis, whereas male Lo KO mice exhibit more fibrosis identifies endogenous Lo FGF2 signaling as desirable for mitigating the effects of cardiac stress while Hi FGF2 signaling is detrimental. Hence, therapeutic targets can be designed to promote Lo FGF2 signaling and block Hi FGF2 signaling in the male heart.

## References

[b1] deAlmeida A, Sedmera D (2009). Fibroblast Growth Factor-2 regulates proliferation of cardiac myocytes in normal and hypoplastic left ventricles in the developing chick. Cardiol. Young.

[b2] Askew GR, Doetschman T, Lingrel JB (1993). Site-directed point mutations in embryonic stem cells: a gene-targeting tag-and-exchange strategy. Mol. Cell. Biol.

[b3] Azhar M, Yin M, Zhou M, Li H, Mustafa M, Nusayr E (2009). Gene targeted ablation of high molecular weight fibroblast growth factor-2. Dev. Dyn.

[b4] Babiker FA, Grohe LJ, De Windt M, Van Eickels C, Meyer R, Doevendans PA (2002). Estrogenic hormone action in the heart: regulatory network and function. Cardiovasc. Res.

[b5] Benjamin IJ, Jalil JE, Tan LB, Cho K, Weber KT, Clark WA (1989). Isoproterenol-induced myocardial fibrosis in relation to myocyte necrosis. Circ. Res.

[b6] Calderone A, Thaik CM, Takahashi N, Chang DL, Colucci WS (1998). Nitric oxide, atrial natriuretic peptide, and cyclic GMP inhibit the growth-promoting effects of norepinephrine in cardiac myocytes and fibroblasts. J. Clin. Invest.

[b7] Coffin JD, Florkiewicz RZ, Neumann J, Mort-Hopkins T, Dorn GW, Lightfoot P (1995). Abnormal bone growth and selective translational regulation in basic fibroblast growth factor (FGF-2) transgenic mice. Mol. Biol. Cell.

[b8] Delrieu I, Arnaud E, Ferjoux G, Bayard F, Faye JC (1998). Overexpression of the FGF-2 24-kDa isoform up-regulates IL-6 transcription in NIH-3T3 cells. FEBS Lett.

[b9] Dixon DW, Loeb HS, Gunnar RM (1979). Use of catecholamines in acute myocardial infarction. Herz.

[b10] Du XJ (2004). Gender modulates cardiac phenotype development in genetically modified mice. Cardiovasc. Res.

[b11] Garmy-Susini B, Delmas E, Gourdy P, Zhou M, Bossard C, Bugler B (2004). Role of fibroblast growth factor-2 isoforms in the effect of estradiol on endothelial cell migration and proliferation. Circ. Res.

[b12] He ZY, Feng B, Yang SL, Luo HL (2005). Intracardiac basic fibroblast growth factor and transforming growth factor-beta 1 mRNA and their proteins expression level in patients with pressure or volume-overload right or left ventricular hypertrophy. Acta Cardiol.

[b13] Hohimer AR, Davis LE, Hatton DC (2005). Repeated daily injections and osmotic pump infusion of isoproterenol cause similar increases in cardiac mass but have different effects on blood pressure. Can. J. Physiol. Pharmacol.

[b14] House SL, House BE, Glascock B, Kimball T, Nusayr E, Schultz JE (2010). Fibroblast growth factor 2 mediates isoproterenol-induced cardiac hypertrophy through activation of the extracellular regulated kinase. Mol. Cell Pharmacol.

[b15] Ibrahimi OA, Zhang F, Eliseenkova AV, Linhardt RJ, Mohammadi M (2004). Proline to arginine mutations in FGF receptors 1 and 3 result in Pfeiffer and Muenke craniosynostosis syndromes through enhancement of FGF binding affinity. Hum. Mol. Genet.

[b16] Levy D, Garrison RJ, Savage DD, Kannel WB, Castelli WP (1990). Prognostic implications of echocardiographically determined left ventricular mass in the Framingham heart study [see comments]. N. Engl. J. Med.

[b17] Liao S, Porter D, Scott A, Newman G, Doetschman T, Schultz JJ (2007). The cardioprotective effect of the low molecular weight isoform of fibroblast growth factor-2: the role of JNK signaling. J. Mol. Cell. Cardiol.

[b18] Liao S, Bodmer JR, Azhar M, Newman G, Coffin JD, Doetschman T (2010). The influence of FGF2 high molecular weight (HMW) isoforms in the development of cardiac ischemia-reperfusion injury. J. Mol. Cell. Cardiol.

[b19] Lorell BH, Carabello BA (2000). Left ventricular hypertrophy: pathogenesis, detection, and prognosis. Circulation.

[b20] Ma X, Dang X, Claus P, Hirst C, Fandrich RR, Jin Y (2007). Chromatin compaction and cell death by high molecular weight FGF-2 depend on its nuclear localization, intracrine ERK activation, and engagement of mitochondria. J. Cell. Physiol.

[b21] Masson S, Arosio B, Luvara G, Gagliano N, Fiordaliso F, Santambrogio D (1998). Remodelling of cardiac extracellular matrix during beta-adrenergic stimulation: upregulation of SPARC in the myocardium of adult rats. J. Mol. Cell. Cardiol.

[b22] Miraoui H, Marie PJ (2010). Fibroblast growth factor receptor signaling crosstalk in skeletogenesis. Sci. Signal.

[b23] Molkentin JD, Dorn IG (2001). Cytoplasmic signaling pathways that regulate cardiac hypertrophy. Annu. Rev. Physiol.

[b24] Nowak HF, Cylwik B, Niczyporuk W (1975). Effect of asparagine and arginine on the repair of isoprenaline-damaged myocardium of rat. Pol. Med. Sci. Hist. Bull.

[b42] Nusayr E, Doetschman T (2013). Cardiac development and physiology are modulated by FGF2 in an isoform- and sex-specific manner. Physiol. Rep.

[b25] Osadchii OE (2007). Cardiac hypertrophy induced by sustained beta-adrenoreceptor activation: pathophysiological aspects. Heart Fail. Rev.

[b26] Padua RR, Kardami E (1993). Increased basic fibroblast growth factor (bFGF) accumulation and distinct patterns of localization in isoproterenol-induced cardiomyocyte injury. Growth Factors.

[b27] Pandya K, Kim HS, Smithies O (2006). Fibrosis, not cell size, delineates beta-myosin heavy chain reexpression during cardiac hypertrophy and normal aging in vivo. Proc. Natl. Acad. Sci. USA.

[b28] Pellieux C, Foletti A, Peduto G, Aubert JF, Nussberger J, Beermann F (2001). Dilated cardiomyopathy and impaired cardiac hypertrophic response to angiotensin II in mice lacking FGF-2. J. Clin. Invest.

[b29] Pintucci G, Yu PJ, Saponara F, Kadian-Dodov DL, Galloway AC, Mignatti P (2005). PDGF-BB induces vascular smooth muscle cell expression of high molecular weight FGF-2, which accumulates in the nucleus. J. Cell. Biochem.

[b30] Prats H, Kaghad M, Prats AC, Klagsbrun M, Lelias JM, Liauzun P (1989). High molecular mass forms of basic fibroblast growth factor are initiated by alternative CUG codons. Proc. Natl. Acad. Sci. USA.

[b31] Quarto N, Fong KD, Longaker MT (2005). Gene profiling of cells expressing different FGF-2 forms. Gene.

[b32] Schluter KD, Zhou XJ, Piper HM (1995). Induction of hypertrophic responsiveness to isoproterenol by TGF-beta in adult rat cardiomyocytes. Am. J. Physiol.

[b33] Schultz JE, Witt SA, Nieman ML, Reiser PJ, Engle SJ, Zhou M (1999). Fibroblast growth factor-2 mediates pressure-induced hypertrophic response. J. Clin. Invest.

[b34] Siri FM (1988). Sympathetic changes during development of cardiac hypertrophy in aortic-constricted rats. Am. J. Physiol.

[b35] Sperinde GV, Nugent MA (2000). Mechanisms of fibroblast growth factor 2 intracellular processing: a kinetic analysis of the role of heparan sulfate proteoglycans. Biochemistry.

[b36] Spruill LS, Baicu CF, Zile MR, McDermott PJ (2008). Selective translation of mRNAs in the left ventricular myocardium of the mouse in response to acute pressure overload. J. Mol. Cell. Cardiol.

[b37] Takeda N, Manabe I (2011). Cellular interplay between cardiomyocytes and nonmyocytes in cardiac remodeling. Int. J. Inflam.

[b38] Touriol C, Bornes S, Bonnal S, Audigier S, Prats H, Prats AC (2003). Generation of protein isoform diversity by alternative initiation of translation at non-AUG codons. Biol. Cell.

[b39] Weber KT, Pick R, Jalil JE, Janicki JS, Carroll EP (1989). Patterns of myocardial fibrosis. J. Mol. Cell. Cardiol.

[b40] Willenbrock R, Stauss H, Scheuermann M, Osterziel KJ, Unger T, Dietz R (1997). Effect of chronic volume overload on baroreflex control of heart rate and sympathetic nerve activity. Am. J. Physiol.

[b41] Yasuno S, Usami S, Kuwahara K, Nakanishi M, Arai Y, Kinoshita H (2009). Endogenous cardiac natriuretic peptides protect the heart in a mouse model of dilated cardiomyopathy and sudden death. Am. J. Physiol. Heart Circ. Physiol.

