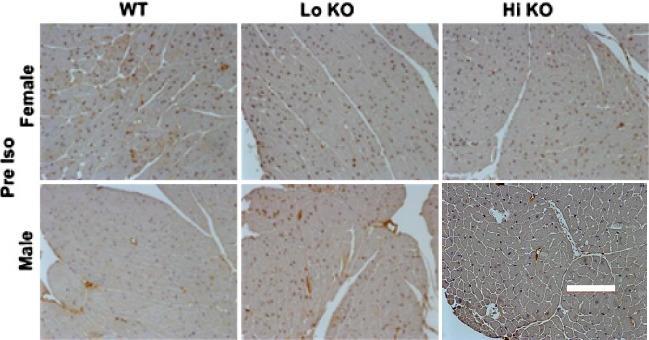# Corrigendum

**DOI:** 10.14814/phy2.12024

**Published:** 2014-06-11

## Introduction

**FGF2 modulates cardiac remodeling in an isoform‐ and sex‐specific manner**

Eyad Nusayr, Doraid Tarek Sadideen & Tom Doetschman

*Physiol Rep*, 1 (4), 2013, e00088, doi: 10.1002/phy2.88

In figure 5, the Hi KO male figure is incorrect. Please refer to the revised figure 5 below.